# Soluble Prion Peptide 107–120 Protects Neuroblastoma SH-SY5Y Cells against Oligomers Associated with Alzheimer’s Disease

**DOI:** 10.3390/ijms21197273

**Published:** 2020-10-01

**Authors:** Elham Rezvani Boroujeni, Seyed Masoud Hosseini, Giulia Fani, Cristina Cecchi, Fabrizio Chiti

**Affiliations:** 1 Department of Microbiology and Microbial Biotechnology, Faculty of Life Science and Biotechnology, Shahid Beheshti University, Tehran 1983969411, Iran; e_rezvaniboroujeni@sbu.ac.ir; 2 Department of Experimental and Clinical Biomedical Sciences, University of Florence, Viale G.B Morgagni 50, 50134 Florence, Italy; giulia.fani@unifi.it (G.F.); cristina.cecchi@unifi.it (C.C.)

**Keywords:** Alzheimer’s disease, prion peptide, Aβ oligomers, ADDLs

## Abstract

Alzheimer’s disease (AD) is the most prevalent form of dementia and soluble amyloid β (Aβ) oligomers are thought to play a critical role in AD pathogenesis. Cellular prion protein (PrP^C^) is a high-affinity receptor for Aβ oligomers and mediates some of their toxic effects. The *N*-terminal region of PrP^C^ can interact with Aβ, particularly the region encompassing residues 95–110. In this study, we identified a soluble and unstructured prion-derived peptide (PrP_107–120_) that is external to this region of the sequence and was found to successfully reduce the mitochondrial impairment, intracellular ROS generation and cytosolic Ca^2+^ uptake induced by oligomeric Aβ_42_ ADDLs in neuroblastoma SH-SY5Y cells. PrP_107–120_ was also found to rescue SH-SY5Y cells from Aβ_42_ ADDL internalization. The peptide did not change the structure and aggregation pathway of Aβ_42_ ADDLs, did not show co-localization with Aβ_42_ ADDLs in the cells and showed a partial colocalization with the endogenous cellular PrP^C^. As a sequence region that is not involved in Aβ binding but in PrP self-recognition, the peptide was suggested to protect against the toxicity of Aβ_42_ oligomers by interfering with cellular PrP^C^ and/or activating a signaling that protected the cells. These results strongly suggest that PrP_107–120_ has therapeutic potential for AD.

## 1. Introduction

Alzheimer’s Disease International (ADI) and the American Alzheimer’s Association (AA) have estimated that over than 50 million people are living with dementia, and that Alzheimer’s disease (AD) is the most common cause and may account for 60–70% of dementia cases [1]. According to this, these numbers will increase dramatically, and this disease will considerably challenge the world healthcare system in the future.

Evidence indicates that AD is an aging-related disease, and frequency in individuals aged 85 or older is higher (one in three) compared to age 65 (one in nine) [1,2]. AD involves a loss of memory, cognitive impairment and behavioral instability [3]. Both extracellular neuritic plaques mainly composed of the amyloid beta (Aβ) peptide and intraneuronal neurofibrillary tangles containing the hyperphosphorylated tau protein are important histopathological hallmarks associated with AD [4,5].

Although accumulation of senile plaques formed by Aβ is a major histopathological trait of AD, it is widely accepted that soluble Aβ oligomers, forming as intermediate species in the process of neuritic plaque formation or released from the plaques, can effectively play a key role in neuronal dysfunction and impair synaptic structure and function [6,7]. It is well-known that such oligomers can inhibit long-term potentiation (LTP)—a correlate of synaptic plasticity [8,9]—as well as activating expression of the complement system [10,11] and general neurotoxic [6,12].

Several studies have reported that the cell membrane protein PrP^C^ mediates the abnormal effects of Aβ oligomers, particularly the oligomer-induced inhibition of LTP [13,14,15]. It was also reported that memory deficits in AD transgenic mice require the presence of PrP^C^ [16], and that loss of synaptic markers, axonal degeneration and early death in transgenic mice are fully dependent on PrP^C^ [17]. Other reports have shown that co-expression of PrP^C^ and Aβ reduces longevity in *Drosophila melanogaster*, and that expression of Aβ individually cannot create pathogenic phenotypes [18]. In spite of the large body of evidence that PrP^C^ mediates the aberrant effects of Aβ oligomers, other studies have indicated that it is not a necessary component for the toxicity cascade induced by Aβ oligomers, as Aβ-induced LTP inhibition or memory impairment has also been reported to occur independently of the overexpression or ablation of PrP^C^ in the transgenic mice [19,20,21]. This has led to a controversy that remains unresolved [22].

PrP^C^ is a membrane protein that is found in both neurons and glial cells [23] and has been proposed to have a role in cellular signaling, copper homeostasis, cell adhesion and even neuroprotection [24,25]. The mature form of human PrP^C^ consists of a single polypeptide chain of 208 amino acid residues (residues 23–230). The *N*-terminal region of the protein (residue 23–120) is unstructured, while the *C*-terminal region (residues 121–230) is largely structured, consisting of three α-helices (residues 144–154, 173–194, 200–228) and two short anti-parallel β-strands (residues 128–131, 161–164) [26]. The protein is anchored to the cell membrane via a glycosyl-phosphatidyl-inositol (GPI) anchor encompassing residues 231–253, which is excised in the mature form [26,27,28]. PrP^C^ function is poorly understood, although roles have been suggested in synaptic transmission, long-term memory, circadian rhythms, T cell function, hematopoietic stem cell renewal, copper-binding, apoptosis and oxidative stress homeostasis, among others [29]. The scrapie form (PrP^Sc^) is derived from PrP^C^ [30,31,32]. The central event is the conversion from an α-helix rich structure to a form with a high β-sheet content. Accumulation of this infectious misfolded form of prion protein (PrP^Sc^) can cause a group of neurodegenerative diseases affecting both human and animals [30,31,32].

A large number of data have shown that PrP^C^ has a high binding affinity for Aβ_42_ oligomers [33,34]. Even studies showing that PrP^C^-expressing and PrP^C^ knock-out mice were equally susceptible to Aβ_42_ oligomer-induced cognitive impairment recognized that the oligomers interacted with PrP^C^ with high affinity [19]. The first evidence of binding between Aβ_42_ oligomers and PrP^C^ dates back to 2009, when it was also found that the *N*-terminal region of PrP^C^, particularly the 95–110 residues, was involved in the binding [35]. One year later, Chen and co-workers showed that both *N*-terminal residues 23–27 and 92–110 were critically important for binding [36]. The importance of these two sequence regions was then confirmed by later reports [13,14,37,38,39,40,41,42].

Unlike the many reports showing a role of the *N*-terminal region of PrP^C^ in the binding of Aβ oligomers, none of the studies reported so far have highlighted a role of the region encompassing residues 106–126 in Aβ_42_ oligomer binding [34]. By contrast, this PrP segment was found to be mainly responsible for prion aggregation [43,44,45,46,47,48,49]. The AGAAAAGA palindromic sequence 113–120, in particular, is necessary for PrP^C^-PrP^Sc^ interaction [44,45], and the two glycine residues at positions 114 and 119 have been suggested as particularly important for fibril formation [50].

As a segment involved in PrP self-recognition, but not in Aβ-PrP^C^ complex formation, we reasoned that a short peptide encompassing this region of the sequence might inhibit Aβ_42_ oligomer toxicity. The hypothesis for this idea is that such a peptide might leave the Aβ oligomers unbound and unaltered, while engaging in interactions with PrP^C^. Our designed peptide encompassing residues 107–120 (PrP_107–120_) was found to be very soluble in physiological conditions, most probably because it lacks the highly hydrophobic region VVGGLG (residues 121–126) of the PrP^C^ fragment 106–126 responsible for prion aggregation [44,45,49]. Importantly, the peptide was found to prevent the generic toxic effects of Aβ_42_ oligomers on neuroblastoma SH-SY5Y cells in absence of Aβ_42_ oligomer binding and structural reorganization, but in presence of partial binding between PrP_107–120_ peptide and endogenous cellular PrP^C^. Since this peptide is not aggregation-prone and has beneficial effects against Aβ-induced toxicity, it is of potentially great interest for rationalizing the pathogenesis of AD and routes to its prevention, as well as in setting up therapeutic strategies for the treatment of AD.

## 2. Results

### 2.1. Freshly Dissolved PrP_107–120_ is Monomeric

**Figure 1:**
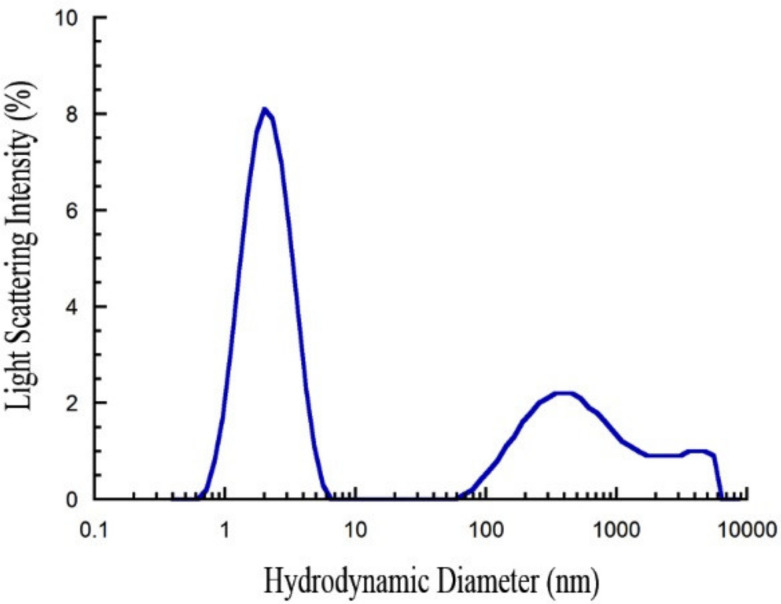
Size distribution of PrP_107–120_. Size distribution by light scattering intensity obtained with dynamic light scattering (DLS) for PrP_107–120_ dissolved in plain water at 25 °C. Peptide concentration was 1 mg/mL. The large aggregates at 70–7000 nm are quantitatively irrelevant, as light scattering intensity scales with the sixth power of the size.

We calculated the theoretical hydrodynamic radius (R_h_) for a peptide with the size of PrP_107–120_ (14 amino acid residues) in an unfolded state, using the equation described in Section 4, and previously published in [51]. According to this equation, the R_h_ value was found to be 0.99 ± 0.56 nm. Dynamic light scattering (DLS) shows that PrP_107–120_ dissolved in water has a hydrodynamic diameter (D_h_) of 2.11 ± 0.68 nm, corresponding to a R_h_ of 1.05 ± 0.34 nm (Figure 1), which is in good agreement with that estimated theoretically. The DLS distribution also showed very large PrP_107–120_ aggregates at 70–7000 nm, but these are quantitatively irrelevant, as light scattering intensity is known to scale with the sixth power of the size. These results reveal that PrP_107–120_ dissolved in water is unfolded and predominantly non-aggregated.

### 2.2. PrP_107–120_ Remains Monomeric and Unstructured under Different Conditions

**Figure 2:**
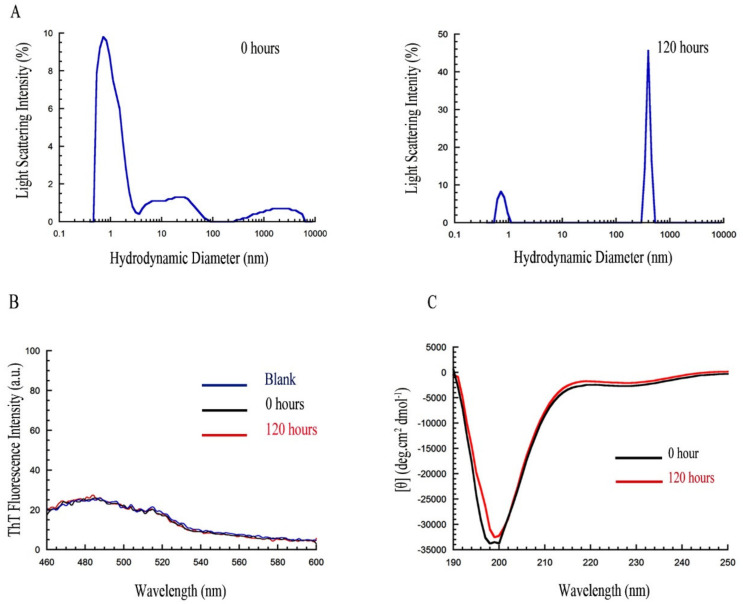
Effect of 20 mM phosphate buffer, 200 mM Na_2_SO_4_, pH 7.0, 37 °C on PrP_107–120_ (1.0 mg/mL) aggregation. (**A**) Size distributions by light scattering intensity of the peptide sample obtained with DLS at *t* = 0 h (left) and 120 h (right) under the conditions described above. (**B**) Thioflavin T (ThT) fluorescence spectra with buffer (blank) and PrP_107–120_ sample after 0 and 120 h. Peptide sample was incubated as described above. ThT assay was carried out at 22 µM ThT, 0.12 mg/mL PrP_107–120_ (final concentrations in the cuvette), pH 6.0, 37 °C. (**C**) Far-UV circular dichroism (CD) spectra of PrP_107–120_ incubated for 0 and 120 h. Peptide sample was incubated as described above and diluted to 0.2 mg/mL in the same buffer before spectrum acquisition at 25 °C.

We then investigated whether different conditions that are generally favorable for protein aggregation have the ability to promote fibrillation for the designed peptide. The different conditions are listed in Section 4 and include various peptide concentrations, salt concentrations, pH values and co-solvents. As a representative example, we show the results obtained at 1.0 mg/mL peptide in 20 mM phosphate buffer, 200 mM Na_2_SO_4_, with a pH 7.0, at 37 °C (Figure 2). The DLS distribution showed a D_h_ of ~1 nm immediately after incubation (0 h) under these conditions, which appears dominant in the population if we take into account that the intensity of scattered light scales with the sixth power of the diameter (Figure 2A). This peak is still clearly visible even after 5 days incubation under these conditions (Figure 2A). Aggregates increased in population after 5 days, yet they appear to be quantitatively irrelevant if we consider the relationship between scattered light intensity and diameter mentioned above. The Thioflavin T (ThT) fluorescence did not increase following the addition of the peptide after 5 days incubation under these conditions relative to the blank containing only ThT (Figure 2B), and the far-UV circular dichroism (CD) spectrum also remained unchanged with a single negative peak at ~198 nm, which is typical of highly disordered states (Figure 2C). Hence, we did not observe a ThT-positive structure or significant changes in size and CD spectrum even after 5 days incubation. Similar results were obtained in all conditions tested (data not shown), indicating that PrP_107–120_ is soluble and stable under all the conditions studied here that are, by contrast, potentially favorable for amyloid fibril formation.

### 2.3. PrP_107-120_ Reduces Aβ_42_ Cytotoxicity on SH-SY5Y Cells

In order to analyze whether PrP_107-120_ can rescue the cellular dysfunction induced by Aβ_42_ oligomers, we used amyloid-derived diffusible ligands (ADDLs) formed from Aβ_42_ peptide according to a well-established protocol [52]. ADDLs were chosen as representative Aβ_42_ oligomers because they are widely used [52,53,54,55], and their morphology and purity are routinely verified [56,57]. These have been found to be toxic and increase intracellular Ca^2+^ and reactive oxygen species (ROS) levels in cultured cells [53,58], and have been found in post-mortem AD brains using both polyclonal and monoclonal conformation-sensitive antibodies specific to ADDLs [54,59]. To this aim, we analyzed the effects of Aβ_42_ ADDLs with a final concentration of 3-µM monomer equivalents (m.e.) on the metabolic activities of human SH-SY5Y cells. This immortalized neuronal cell model is mostly used for AD research, as human cholinergic neurons are difficult to obtain and maintain and unsuitable for routine experiments.

**Figure 3:**
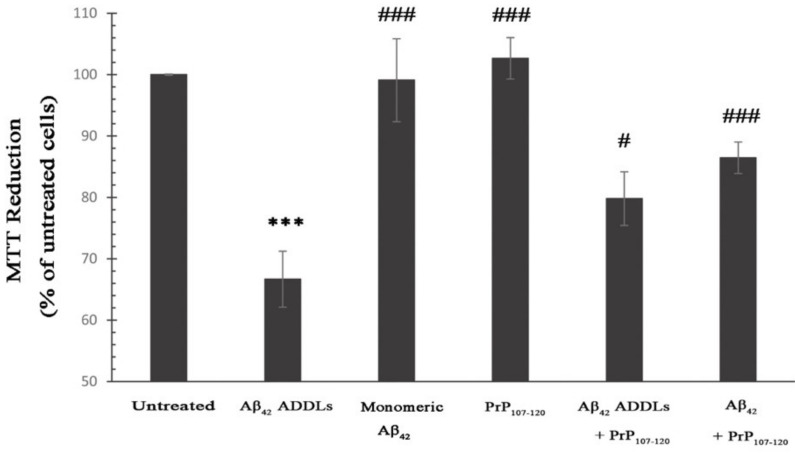
Cell viability assay in the presence of Aβ_42_ oligomers and PrP_107–120_. 3-(4,5-dimethylthiazol-2-yl)-2,5-diphenyltetrazolium bromide (MTT) reduction capacity of SH-SY5Y cells following 24 h treatment with Aβ_42_ amyloid-derived diffusible ligands (ADDLs), monomeric Aβ_42_, PrP_107–120_, Aβ_42_ ADDLs + PrP_107–120_ and Aβ_42_ and PrP_107–120_ pre-incubated under conditions promoting ADDL formation prior to addition to the cell medium. All samples were initially in 2% (*v*/*v*) dimethyl sulfoxide (DMSO) and F-12 Ham medium at concentrations of 100 and 25 µM (m.e.) for Aβ_42_ and PrP_107–120_, respectively, and were diluted 33-fold before each experiment into cellular medium without DMSO to final concentrations of 3 and 0.75 µM (m.e.) for Aβ_42_ and PrP_107–120_, respectively. The data shown are mean values ± SEM of five independent experiments. The triple asterisks (***) refer to *p* values < 0.001 relative to the untreated cells. The single (#) and triple (###) symbols refer to *p* values < 0.05 and < 0.001, respectively, relative to Aβ_42_ ADDLs.

The metabolic activity of the cells was analyzed using the 3-(4,5-dimethylthiazol-2-yl)-2,5-diphenyltetrazolium bromide (MTT) assay, which is a widely used indicator of mitochondrial reduction capacity. The ability of SH-SY5Y cells to reduce MTT significantly decreased to 66.6 ± 4.5% following treatment for 24 h with Aβ_42_ ADDLs at 3 µM m.e. (Figure 3). By contrast, no detectable change was observed in cells treated with PrP_107–120_ and monomeric Aβ_42_ at final concentrations of 0.75 and 3 µM, respectively (Figure 3). The addition of the PrP_107–120_ (0.75 µM) to Aβ_42_ ADDLs (3 µM) significantly increased cell viability to 79.7 ± 4.3% compared with the cells treated with Aβ_42_ ADDLs (3 µM) in the absence of the peptide (Figure 3). In another experiment, PrP_107–120_ at a concentration of 0.75 µM was added to monomeric Aβ_42_ peptide at a concentration of 3 µM, and the resulting sample was maintained under conditions favorable for ADDL formation before addition to the cells (Aβ_42_ + PrP_107–120_). In this case, we found a cell viability of 86.4 ± 2.5%, again indicating protection by the PrP_107–120_ peptide. These results suggest that PrP_107–120_ can act as an inhibitor of Aβ_42_ ADDL oligomer toxicity.

### 2.4. PrP_107-120_ Reduces Ca^2+^ Influx Induced by Aβ_42_ Oligomers

**Figure 4:**
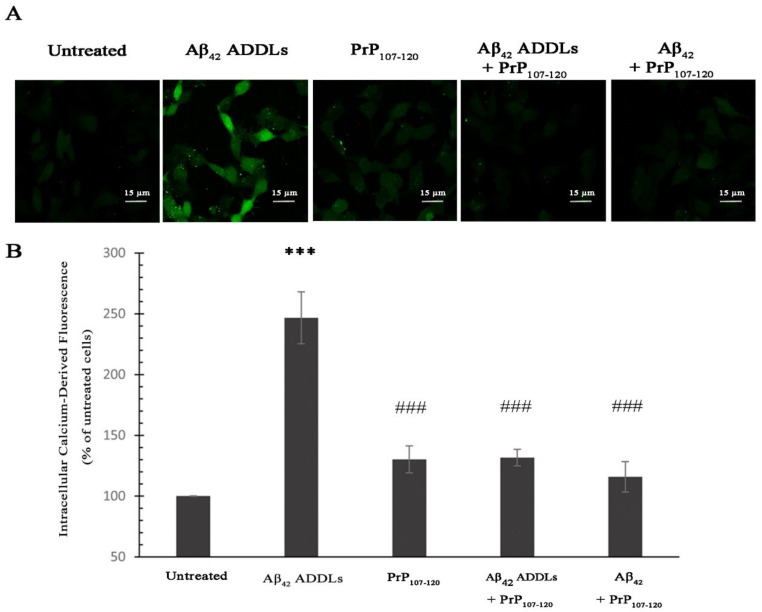
Analysis of intracellular Ca^2+^ levels of SH-SY5Y cells treated with Aβ_42_ oligomers and PrP_107–120_. (**A**) Representative scanning confocal microscopy images of intracellular free Ca^2+^ levels in SH-SY5Y cells loaded with Fluo-4 AM probe. The cells were treated for 1 h with Aβ_42_ ADDLs, PrP_107–120_, Aβ_42_ ADDLs + PrP_107–120_ and Aβ_42_ + PrP_107–120_ pre-incubated under conditions promoting ADDL formation prior to addition to the cell medium. All samples were initially in 2% (*v*/*v*) DMSO and F-12 Ham medium at concentrations of 100 and 25 µM (m.e.) for Aβ_42_ and PrP_107–120_, respectively, and were diluted 33-fold before each experiment into cellular medium without DMSO to final concentrations of 3 and 0.75 µM (m.e.) for Aβ_42_ and PrP_107–120_, respectively. Scale bar = 15 µm. **(B**) Semi-quantitative analysis of intracellular Ca^2+^ derived fluorescence. Experimental errors are S.E.M. (*n* = 4). The triple (***) asterisks refer to *p* values < 0.001 relative to the untreated cells. The triple (###) symbols refer to *p* values < 0.001 relative to Aβ_42_ ADDLs.

An early cellular insult caused by Aβ_42_ ADDLs, and Aβ_42_ oligomers more generally, when added to the cellular medium, is the permeabilization of the plasma membrane with a rapid influx of calcium ions (Ca^2+^) from the extracellular space to the cytosol [53,58,60]. To investigate whether PrP_107–120_ can prevent the increase of Ca^2+^ levels mediated by the Aβ_42_ ADDL oligomers, we monitored the influx of Ca^2+^ in SH-SY5Y cells treated with Aβ_42_ ADDLs in presence and absence of PrP_107–120_, at the same concentrations used for the MTT assay. The quantification of the intracellular Ca^2+^-derived fluorescence in confocal microscopy images shows that the treatment of the cells with Aβ_42_ ADDLs caused a significant increase in intracellular Ca^2+^ up to 246 ± 21% compared with untreated cells, taken as 100% (Figure 4). By contrast, the cellular exposure to Aβ_42_ + PrP_107–120_, Aβ_42_ ADDLs + PrP_107–120_ and PrP_107–120_ alone triggered a minor increase of intracellular Ca^2+^-derived fluorescence (i.e., to 116 ± 13%, 132 ± 7% and 130 ± 11% respectively) that was significantly lower than that observed in cells treated with Aβ_42_ ADDLs (Figure 4). This result suggests a protective role of PrP_107–120_ in the Ca^2+^ influx mediated by the Aβ_42_ oligomers in neuronal cells. Since the concentrations of Ca^2+^ and PrP_107–120_ in the cell medium were 2 mM and 0.75 µM, respectively, it can be ruled out that the inhibition of ADDL-induced Ca^2+^ influx mediated by the prion peptide arises from peptide-Ca^2+^ binding, as the peptide is over three orders of magnitude sub-stoichiometric.

### 2.5. PrP_107-120_ Reduces Intracellular Reactive Oxygen Species (ROS) Induced by Aβ_42_ Oligomers

**Figure 5:**
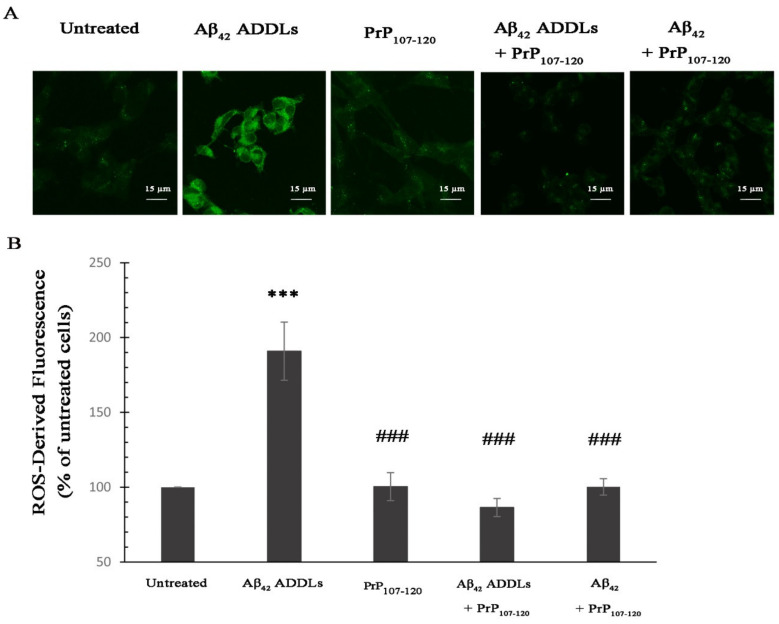
Analysis of intracellular reactive oxygen species (ROS) levels of SH-SY5Y cells treated with Aβ_42_ oligomers and PrP_107–120_. (**A**) Representative scanning confocal microscopy images of intracellular free ROS levels in SH-SY5Y cells loaded with CM-H_2_DCFDA. The cells were treated with the same samples described in the Figure 4 legend. Scale bar = 15 µm. (**B**) Semi-quantitative analysis of intracellular ROS-derived fluorescence. Experimental errors are S.E.M. (*n* = 3). The triple (***) asterisks refer to *p* values < 0.001 relative to the untreated cells. The triple (###) symbols refer to *p* values < 0.001 relative to Aβ_42_ ADDLs.

Another effect caused early by Aβ_42_ ADDLs when added to the extracellular medium of cells is the increase of ROS levels in the cytosol [58,61]. We observed an increase of ROS-derived fluorescence up to 190 ± 19% in SH-SY5Y cells treated with Aβ_42_ ADDLs compared with untreated cells, and no significant change in cells treated with PrP_107–120_ alone (Figure 5). By contrast, the treatment with Aβ_42_ ADDLs + PrP_107–120_ and Aβ_42_ + PrP_107–120_ did not cause any significant change in ROS-derived fluorescence levels compared with untreated cells, and levels significantly lower than those observed after treatment with Aβ_42_ ADDLs (Figure 5). Therefore, PrP_107–120_ can effectively protect SH-SY5Y cells against the oxidative stress induced by Aβ_42_ ADDLs.

### 2.6. PrP_107-120_ Reduces the Toxicity of Other Model Oligomers

**Figure 6:**
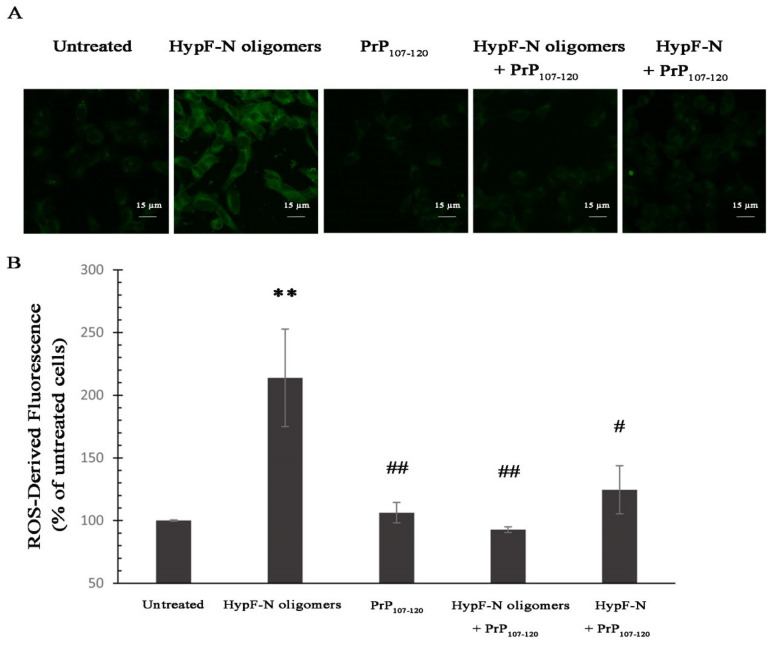
Analysis of intracellular ROS levels of SH-SY5Y cells treated with type A HypF-N oligomers and PrP_107–120_. (**A**) Representative scanning confocal microscopy images of intracellular free ROS levels in SH-SY5Y cells loaded with CM-H_2_DCFDA. The cells were treated for 1 h with HypF-N oligomers, PrP_107–120_, HypF-N oligomers + PrP_107-120_ and a sample containing HypF-N and PrP_107–120_ pre-incubated under conditions promoting HypF-N oligomer formation prior to addition to the cell medium. Final HypF-N and PrP_107–120_ concentrations were 12 and 3 µM (m.e.), respectively. Scale bar = 15 µm. (**B**) Semi-quantitative analysis of intracellular ROS-derived fluorescence. Experimental errors are S.E.M. (*n* = 3). The double (**) asterisks refer to *p* values < 0.01 relative to the untreated cells. The double (##) and single (#) symbols refer to *p* values < 0.01 and < 0.05, respectively, relative to HypF-N oligomers.

In order to assess whether the protective role of PrP_107–120_ observed with ADDLs was specific to this peptide and oligomer system or was more generally exerted against misfolded protein oligomers, we also tested whether PrP_107–120_ has the ability to decrease the cytotoxicity of another type of misfolded oligomer, those formed by the model protein HypF-N named type A and found to have effects similar to those of Aβ_42_ [62,63,64,65]. We observed an increase of ROS-derived fluorescence in SH-SY5Y cells upon treatment with type A HypF-N oligomers up to 214 ± 39% (Figure 6). Exposure to HypF-N oligomers + PrP_107–120_ and HypF-N preincubated with PrP_107–120_ under conditions promoting type A HypF-N oligomer formation showed non-significant changes in intracellular ROS-derived fluorescence (93 ± 2% and 124 ± 19%, respectively).

### 2.7. PrP_107-120_ Reduces Aβ_42_ ADDLs Internalization in SH-SY5Y Cells

**Figure 7:**
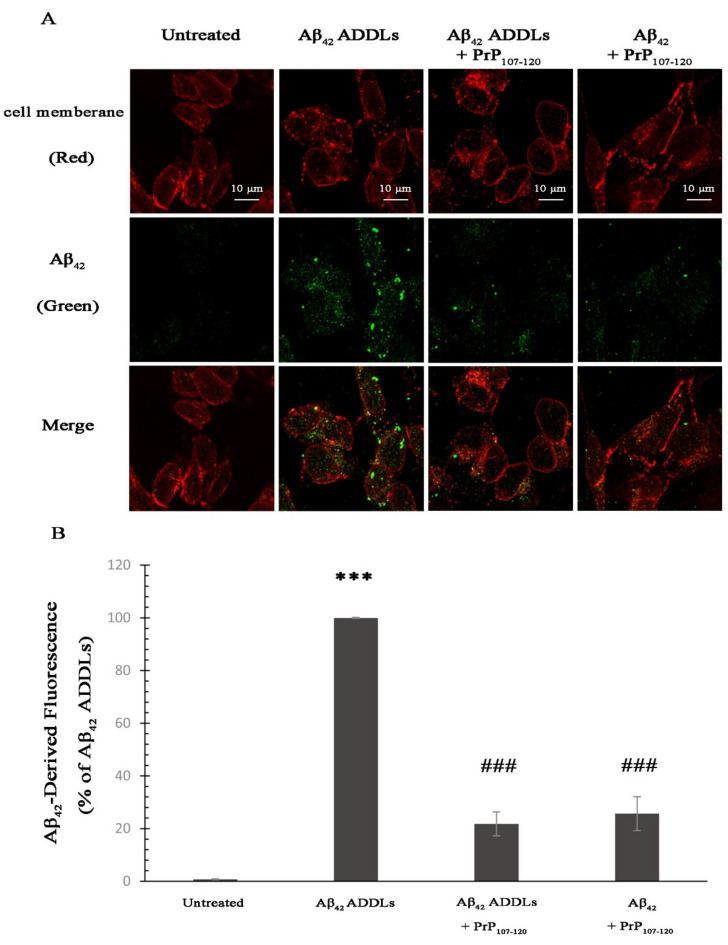
Effect of PrP_107–120_ on Aβ_42_ ADDL internalization in SH-SY5Y cells. (**A**) Representative scanning confocal microscopy images of SH-SY5Y cells treated with the indicated samples and showing Aβ_42_ ADDLs. The cells were treated with the same samples described in the Figure 4 legend. The cellular membrane was stained with wheat germ agglutinin (red fluorescence) and Aβ_42_ ADDLs were labelled with mouse monoclonal primary antibody 6E10 and anti-mouse secondary antibody (green fluorescence). Scale bar = 10 µm. (**B**) Semi-quantitative analysis of intracellular Aβ_42_ ADDL-derived fluorescence. Experimental errors are S.E.M. (*n* = 3). The triple (***) asterisks refer to *p* values < 0.001 relative to the untreated cells. The triple (###) symbols refer to *p* values < 0.001 relative to Aβ_42_ ADDLs.

In order to study the effects of PrP_107-120_ on the cellular internalization of Aβ_42_ ADDLs in SH-SY5Y cells, we performed immunostaining experiments using the 6E10 specific antibody against Aβ_42_ and wheat germ agglutinin to stain Aβ_42_ and the cell membrane, respectively. Cells were exposed for 1 h to the various protein samples described above and added to the cell medium. Thus, for the intracellular Aβ_42_-derived fluorescence (green) in the SH-SY5Y cells, we observed more than 78% reduction in cells treated with Aβ_42_ ADDLs + PrP_107–120_, and more than 74% reduction in cells treated with Aβ_42_ + PrP_107–120_ in terms of intracellular ADDLs levels with respect to cells treated with Aβ_42_ ADDLs taken as 100% (Figure 7). These results indicate that PrP_107–120_ is able to significantly reduce Aβ_42_ ADDL internalization in SH-SY5Y cells when added either before or after the Aβ_42_ oligomerization process.

### 2.8. PrP_107-120_ Does Not Change the Structure or Aggregation State of Aβ_42_ ADDLs

To determine whether PrP_107–120_ can modify the structure of Aβ_42_ ADDLs into non-toxic Aβ_42_ oligomers or cause a change in their aggregation state (either fibrils, large aggregates or monomers), we carried out a number of tests using dot-blot, ThT fluorescence, far-UV CD and ANS fluorescence on ADDLs in the presence and absence of PrP_107–120_, using the same samples used for cell toxicity.

**Figure 8:**
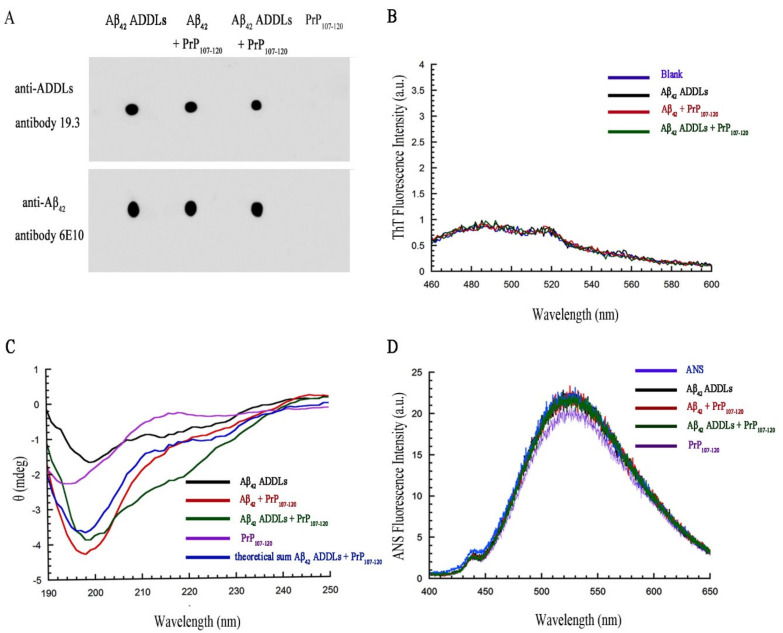
Effect of PrP_107–120_ on Aβ_42_ ADDL structure. (**A**) Dot-blot immunoassay for Aβ_42_ ADDLs, Aβ_42_ + PrP_107–120_, Aβ_42_ ADDLs + PrP_107–120_ and PrP_107–120_ samples. All samples were initially in 2% (*v*/*v*) DMSO and F-12 Ham medium at concentrations of 100 and 25 µM (m.e.) for Aβ_42_ and PrP_107–120_, respectively. They were then spotted in two different nitrocellulose membranes, probed with the conformation-sensitive anti-ADDL antibody 19.3 (first line) and with conformation-insensitive anti-Aβ_42_ antibody 6E10 (second line). (**B**) ThT fluorescence assay for free ThT (blue), Aβ_42_ ADDLs (black), Aβ_42_ + PrP_107–120_ (red) and Aβ_42_ ADDLs + PrP_107–120_ (green). Samples were incubated as described above. ThT assay was carried out at 22 µM ThT (final concentration in the cuvette), pH 6.0, 37 °C. (**C**) Far-UV CD spectra for Aβ_42_ ADDLs (black), Aβ_42_ + PrP_107–120_ (red) and Aβ_42_ ADDLs + PrP_107–120_ (green) and PrP_107–120_ (purple). Samples were incubated as described above and diluted before spectrum acquisition to final concentrations of 22.2 µM Aβ_42_ and 5.55 µM PrP_107–120_, 25 °C. (**D**) ANS fluorescence spectra for free ANS (55 µM final concentration) and the same samples indicated in panel C. Fluorescence spectra were recorded at 25 °C.

The presence of Aβ_42_ ADDLs was monitored by a dot-blot immunoassay using the conformation-sensitive antibody 19.3 specific for Aβ_42_ ADDLs [66] and the monoclonal antibody 6E10, which is able to bind all types of Aβ_42_ species. For Aβ_42_ ADDLs, Aβ_42_ + PrP_107–120_ and Aβ_42_ ADDLs + PrP_107–120_ we observed a recognition for both antibodies, whereas the PrP_107–120_ spot did not show any cross-reaction (Figure 8A), suggesting that PrP_107–120_ cannot change the structure or oligomerization state of ADDLs.

Aβ_42_ ADDLs did not bind ThT and did not increase its fluorescence, behavior that was not found to be affected by the PrP_107–120_ peptide (Figure 8B) and suggests that the peptide was not able to change the structure of the ADDLs into a stable and ThT-positive β-sheet structure. Moreover, the CD spectra observed for the Aβ_42_ + PrP_107–120_ and Aβ_42_ ADDLs + PrP_107–120_ were found to be similar to those resulting from the sum of the spectra of Aβ_42_ ADDLs alone and PrP_107–120_ alone, indicating that the PrP_107–120_ peptide did not significantly change the secondary structure of Aβ_42_ ADDLs (Figure 8C). Finally, the spectrum of ANS in the presence of Aβ_42_ ADDLs did not change if the ADDLs were formed in the presence of the peptide (Aβ_42_ + PrP_107–120_) or pre-incubated in the presence of the peptide after their formation (Aβ_42_ ADDLs + PrP_107–120_), suggesting again that the PrP_107–120_ peptide was not able to change the Aβ_42_ ADDL structure (Figure 8D).

### 2.9. PrP_107-120_ Does Not Colocalise with Aβ_42_ ADDLs but Partially Colocalises with Cellular PrP^C^ in SH-SY5Y Cells

**Figure 9:**
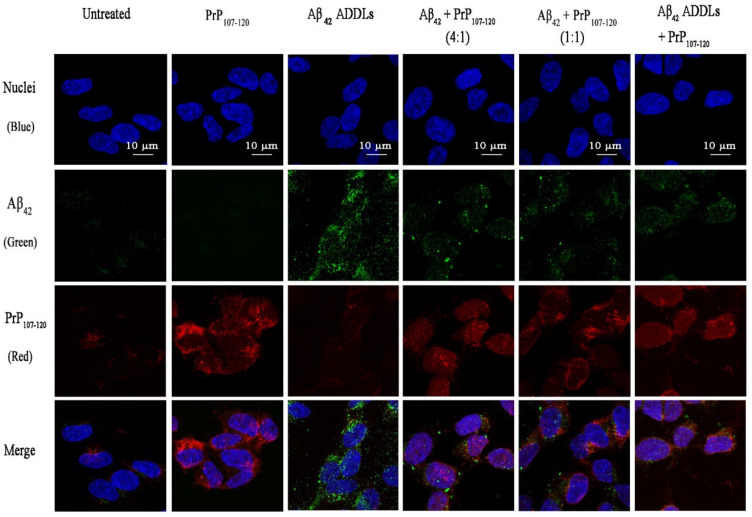
Absence of co-localization of Aβ_42_ ADDLs with PrP_107–120_. Cells were treated for 1 h with the same samples described in the Figure 4 legend and indicated on top of the images (added to the cell medium). PrP_107–120_ was labelled with BODIPY TMR-X NHS Ester (red fluorescence), Aβ_42_ ADDLs with mouse monoclonal primary antibody 6E10 and a secondary antibody (green fluorescence) and the nuclei with the Hoechst dye (blue florescence). Scale bar = 10 µm.

**Figure 10:**
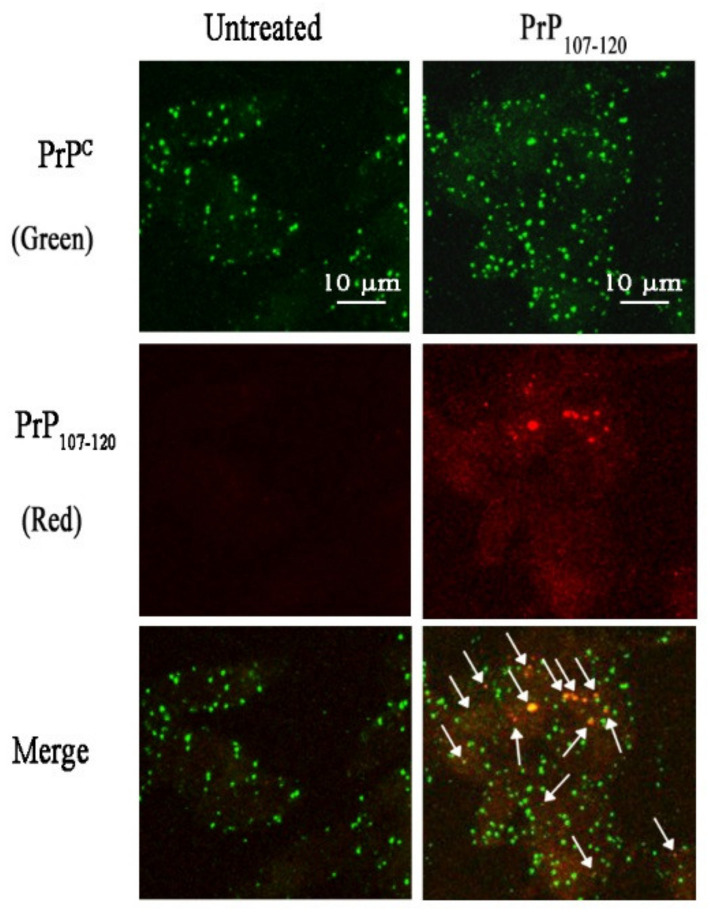
Partial co-localization of PrP_107-120_ with endogenous cellular PrP^C^. Cells were treated for 1 h with the PrP_107–120_ sample described in the Figure 9 legend and indicated on top of the images (added to the cell medium). PrP_107–120_ was labelled with BODIPY TMR-X NHS Ester (red fluorescence) and cellular PrP^C^ was labelled with mouse monoclonal primary antibody PrP (5B2) and Alexa Fluor 488-conjugated anti-mouse secondary (green fluorescence). The white arrows indicate the co-localization spots in the merged images. Scale bar = 10 µm.

The inability of PrP_107-120_ to change the structure of Aβ_42_ ADDLs, yet its ability to decrease ADDL toxicity, raised two possible hypotheses: (i) PrP_107-120_ binds to ADDLs and shields their hydrophobic patches that are responsible for toxicity, or (ii) it interacts with the cells and renders them less vulnerable to ADDL toxicity. To verify which hypothesis was correct, the interaction of PrP_107–120_ and Aβ_42_ was studied in cell cultures. Confocal microscopy images showed an absence of co-localization between Aβ_42_ ADDLs (3 µM m.e.) and PrP_107-120_, both with ratios of 4:1 (0.75 µM PrP_107–120_) and 1:1 (3 µM PrP_107–120_), suggesting an absence of interaction between the two peptides (Figure 9). By contrast, PrP_107–120_ (0.75 µM) was found to partially colocalize with endogenous cellular PrP^C^ (Figure 10, white arrows). These results also showed a significant, albeit weak, expression of PrP^C^ in our SH-SY5Y cell system, which is in agreement with the levels of expression of the Human Protein Atlas (Figure 10).

## 3. Discussion

The results obtained here show that the synthetic peptide PrP_107–120_ is soluble and unstructured in solution and can significantly protect neuroblastoma SH-SY5Y cells against a representative form of oligomeric Aβ_42_, namely, ADDLs. In particular, in the presence of the peptide, Aβ_42_ oligomers caused a remarkably lower Ca^2+^ influx from the cellular medium to the cytosol, a remarkably lower increase of cellular ROS and a significantly lower alteration of mitochondrial metabolic activity. Furthermore, they had a remarkably lower ability to enter the cytosol across the cell membrane. The absence of interaction of the PrP_107–120_ peptide with ADDLs, and the partial colocalization of the peptide with endogenous cellular PrP^C^, indicates that the peptide protects the cells against Aβ_42_ oligomer toxicity, at least in part, by interfering with cellular PrP^C^.

Previous studies have suggested that the cell surface protein PrP^C^ mediates the toxicity of Aβ_42_ oligomers by binding to them and affecting synaptic plasticity and other neuronal functions [13,14,15,16,17,18], although a general consensus on this point has not yet been found [19,20,21]. One PrP^C^ region of the sequence thought to be involved in Aβ_42_ binding encompasses approximately residues 95–110 [13,14,33,34,35,37,38,39,40,41,42]. An antibody raised against residues 93–109 of PrP^C^ (anti-PrP^C^_93–109_ GD11) and a synthetic peptide corresponding to residues 98–107 (PrP_98–107_) were found to inhibit the toxicity of the Aβ_42_ ADDLs to organotypic hippocampal slices, whereas a control PrP_213–230_ peptide did not have any such effects [37]. It was proposed that the antibody and the PrP_98–107_ peptide exert their effect by binding to PrP^C^ and the diffusible Aβ_42_ oligomers, respectively, thus preventing their interaction in both cases [37].

In this study we have used a different perspective: Rather than using a synthetic PrP^C^ peptide binding to Aβ_42_, we have used a synthetic PrP^C^ peptide involved in PrP self-recognition (PrP_107–120_), with the goal of achieving a similar biological result with a different mechanism, namely, binding to membrane-anchored PrP^C^ and impeding the binding of the latter to Aβ_42_ oligomers. We found that PrP_107–120_ significantly reduced the toxicity of Aβ_42_ oligomers with or without pre-incubation of the peptide with Aβ_42_ during the process of ADDL oligomer formation. Biophysical analyses and a dot-blot immunoassay excluded an alteration of the Aβ_42_ ADDL structure or aggregation state in the presence of the peptide, ruling out the hypothesis that the reduction of Aβ_42_ oligomer toxicity and internalization mediated by PrP_107-120_ is due to this reason. Moreover, no PrP_107–120_ colocalization with Aβ_42_ was found in the cell cultures using confocal microscopy even at high concentrations of PrP_107–120_, indicating a lack of any significant interaction between the two peptide species. In contrast, a partial colocalization between PrP_107-120_ and endogenous PrP^C^ was found, indicating that the peptide can bind to the cellular prion protein. Furthermore, akin to previous observations, the role of PrP^C^ in mediating oligomer toxicity is not restricted to Aβ_42_ oligomers, but also to other β-sheet rich proteins [67], and our results indicate a similar effect of PrP_107–120_ on Aβ_42_ oligomers and type-A HypF-N oligomers, used here as a positive control of toxic oligomeric species. However, we cannot completely exclude that PrP_107–120_ stimulates a well-defined intracellular signaling [25] and causes less vulnerability and greater resistance against Aβ_42_ oligomer toxicity independently of its interaction with membrane-anchored PrP^C^.

Overall, although the results obtained here in vitro on a cell culture line need to be validated in vivo on animal models, the present study shows the potential therapeutic value of peptides corresponding to the region of the sequence of PrP^C^ involved in self-recognition, or other molecules potentially mimicking the same sequence trait, for the treatment of AD. This could open new avenues to the identification of the mechanism of interaction of soluble prion-derived peptides and Aβ_42_ oligomers, as well as to the mechanism through which PrP^C^ mediates the toxic effects of Aβ_42_ oligomers.

## 4. Materials and Methods

### 4.1. PrP_107–120_ Preparation

The synthetic human prion protein fragment spanning residues 107–120 (PrP_107–120_) with the sequence Ac-TNMKHMAGAAAAGA (purity by HPLC > 95%) was purchased from Biomatik (Wilmington, DE, USA). The <5% impurities are mainly peptides with similar sequences, as are often found in solid-state peptide synthesis [68]. The counterions of peptide preparations (trifluoroacetate and guanidinium) are not supposed to be cell protectors and did not interfere with our analysis. Metal ions or other agents were not present. The lyophilized peptide was stored at −20 °C, and for each experiment 1 mg of peptide was dissolved in 1 mL of water.

### 4.2. Preparation of Aβ_42_ ADDLs, Aβ_42_ ADDLs + PrP_107–120_ and Aβ_42_ + PrP_107–120_ Samples

Lyophilized synthetic Aβ_42_ in a trifluoroacetate salt (Bachem, Bubendorf, Switzerland) was dissolved in pure hexafluoro-2-isopropanol (HFIP) to 1 mM. For each experiment, the solvent was evaporated using gentle nitrogen flow and the peptide was reconstituted in 2% (*v*/*v*) dimethyl sulfoxide (DMSO) and F12 Ham medium to a final concentration of 100 µM. Aβ-derived diffusible ligands (ADDLs) were prepared after 24 h incubation at 4 °C, as previously reported [52], and checked for their distinctive characteristics using atomic force microscopy, Western blotting and dot-blot, as previously described [56,57]. In this study, we used the following samples: (A) 100 µM m.e. of Aβ_42_ ADDLs; (B) 25 µM PrP_107–120_ (final concentration) added to preformed 100 µM m.e. of Aβ_42_ ADDLs at a 1:4 molar ratio, incubated at 4 °C for 2 h (Aβ_42_ ADDLs + PrP_107–120_); (C) 25 µM PrP_107–120_ (final concentration) added to 100 µM Aβ_42_ at a 1:4 molar ratio before Aβ_42_ aggregation, maintained at 4 °C for 24 h under the same conditions used to form Aβ_42_ ADDLs (Aβ_42_ + PrP_107–120_); and (D) 25 µM PrP_107–120_. All four samples were in 2% (*v*/*v*) DMSO and F-12 Ham medium and were diluted 33-fold before each experiment into cellular medium without DMSO to final concentrations of 3 and 0.75 µM (m.e.) for Aβ_42_ and PrP_107–120_, respectively.

### 4.3. Preparation of HypF-N Oligomers

HypF-N was purified as described previously [62] and stored at −80 °C. Before each experiment, the protein sample was thawed, centrifuged at 13,000 rpm (17950× *g*) for 10 min, and the concentration was measured at 280 nm. The sample was then diluted to 48 µM in 12% (*v*/*v*) trifluoroethanol, 50 mM acetate buffer and 2 mM dithiothreitol, at pH 5.5, as this condition is known to promote aggregation of HypF-N and proteins of the same structural family [62,69]. After 4 h at 25 °C, the sample was centrifuged at 12,000 rpm (15300× *g*) for 15 min and the pellet was dried with a gentle nitrogen flow and resuspended to 12 µM m.e. in cellular medium with or without PrP_107–120_, to a final concentration of 3 µM (4:1 molar ratio).

### 4.4. Bicinchoninic Acid (BCA) Assay

Stock bovine serum albumin (BSA) standard (Sigma-Aldrich, Saint Louis, MO, USA) was prepared at a 2-mg/mL final concentration in water. Eight BSA samples with concentrations ranging from 0 to 2000 µg/mL were prepared by dilution for the standard curve. The bicinchoninic acid (BCA) working reagent was prepared by mixing 10 mL of solution A (Bicinchoninic Acid solution, Sigma-Aldrich) with 200 µL of solution B (4% *w*/*v* CuSO_4_·5H_2_O in water). The blank and protein samples were mixed with the resulting BCA working reagent at a 1:8 ratio; thus, 25 µL of each peptide or BSA or blank samples were mixed with 200 µL of BCA working reagent and were added to 96-microplate wells. The plate was incubated at 60 °C for 15 min and, after 5 min cooling to room temperature, the absorbance of all wells was measured at 562 nm using an ultrafast BioTek Synergy H1 plate reader (Winooski, VT, USA). All absorbance values were blank subtracted. Peptide concentration was calculated by interpolation using a standard curve (absorbance versus protein concentration) obtained with the eight BSA samples.

### 4.5. Fibrillation of PrP_107–120_

The aggregation kinetics of PrP_107–120_ was investigated under different solution conditions at 37 °C: (A) 0.5 mg/mL peptide in 20 mM phosphate buffer, 200 mM NaCl, pH 7.0; (B) 0.5 mg/mL peptide in 20 mM HCO_3_^–^, 200 mM NaCl, pH 10.5; (C) 0.5 mg/mL peptide in 20 mM phosphate buffer, 200 mM NaCl, 10% (*v*/*v*) trifluoroethanol, pH 7.0; (D) 1.0 mg/mL peptide in 20 mM acetate buffer, 200 mM NaCl, pH 4.0; (E) 1.0 mg/mL peptide in 20 mM HCO_3_^–^, 200 mM NaCl, pH 10.5; (F) 1.0 mg/mL peptide in 20 mM phosphate buffer, 500 mM NaCl, pH 7.0; (G) 1.0 mg/mL peptide in 20 mM phosphate buffer, 15% (*v*/*v*) methanol, pH 7.0; (H) 1.0 mg/mL peptide in 20 mM phosphate buffer, 200 mM Na_2_SO_4_, pH 7.0. Fibril formation was monitored by the ThT fluorescence assay, DLS and CD spectroscopy for all conditions.

### 4.6. Dynamic Light Scattering

In a first experiment, to determine the size of monomeric PrP_107–120_, 1 mg of peptide was dissolved in 1 mL of water. The sample was filtered through a 0.22-µm filter and size distribution analysis was performed at 25 °C by a Zetasizer Nano S DLS device from Malvern Panalytical (Malvern, Worcestertshire, UK) thermostated with a Peltier system and checked for its reliability with polystyrene latex beads with known hydrodynamic diameter. A 10-mm plastic cell with a reduced volume was used. The refractive index and viscosity were 1.333 and 0.88 cP. The cell position and attenuator index were set automatically. The theoretical R_h_ for a peptide was calculated by the following equation:

R_h_ (Å) = (2.21 ± 1.07) N ^0.57 ± 0.02^ (1)

where N is the number of amino acid residues [46]. The theoretical D_h_ was twice the R_h_ value. In a second experiment, to investigate the effect of different conditions on peptide aggregation, measurements were carried out using 1 mg of peptide dissolved in 1 mL of selected buffers and following the size distribution over time for several days at 37 °C, using the same technical apparatus and cell described above. The refractive index and viscosity were set according to the various conditions.

### 4.7. ThT Fluorescence

ThT (Sigma-Aldrich) was dissolved to 25 µM, in 25 mM phosphate buffer, with a pH of 6.0, then filtered by a 0.45-µm filter. For a given peptide sample, 60 µL aliquots were added to 440 µL of resulting solution. A 2 × 10 mm optical path-length cuvette was used, and fluorescence spectra were recorded at 37 °C using a PerkinElmer LS 55 fluorimeter (Waltham, MA, USA) equipped with a thermostated cell holder attached to a Thermo Haake C25P water bath (Karlsrube, Germany) with an excitation wavelength of 440 nm and an emission wavelength range of 460–600 nm.

### 4.8. Circular Dichroism (CD) Spectroscopy

To determine the PrP_107-120_ polymerization kinetics in the presence of different conditions, 0.2 mg/mL of PrP_107-120_ peptide was used. To investigate the effect of the PrP_107–120_ peptide on Aβ_42_ oligomeric ADDLs, three samples were prepared, each 300 µL, including two separate samples of 22.2 µM Aβ_42_ ADDLs and one sample containing both 22.2 µM Aβ_42_ and 5.55 µM PrP_107–120_ at a 4:1 molar ratio. For removing DMSO, dialysis was performed using Spectra/Por 3 dialysis kits (MWCO 3.5 kDa, Spectrum Labs/Thermo Fisher Scientific, Waltham, MA, USA) and 10 mM phosphate buffer, with a pH of 6.0, for 3 h at 4 °C. For the preparation of Aβ_42_ ADDLs + PrP_107–120_ sample, PrP_107–120_ was added at a final concentration of 5.55 µM after dialysis and was incubated for 2 h at 4 °C. Far-UV CD spectra were measured at 25 °C between 190–260 nm with a 1-nm spectral step size and 50-nm/min scanning rate on a JASCO J-810 spectropolarimeter (Tokyo, Japan) equipped with thermostated cell-holder attached to a Thermo Haake C25P water bath (Karlsruhe, Germany). A 1-mm thermostated quartz cuvette was used for all CD spectra. Spectra were blank-subtracted and normalized to mean residue ellipticity.

### 4.9. Cell Culture

Adherent human neuroblastoma SH-SY5Y cells (A.T.C.C, Manassas, VA, USA) were cultured in Dulbecco’s Modified Eagle’s Medium (DMEM, Sigma-Aldrich), F-12 Ham with 25 mM n-(2-hydroxyethyl)piperazine-n’-(2-ethanesulfonic) acid (HEPES) and NaHCO_3_, supplemented with 10% fetal bovine serum (FBS), 2 mM glutamine and 1% penicillin/streptomycin and maintained at 37 °C and 5% CO_2_. When the cells reached 90% confluence, they were split using 0.25% trypsin-EDTA to a maximum of 20 passages.

### 4.10. MTT Assay

SH-SY5Y cells were plated at a density of 15 × 10^3^ cells per well in a 96-well plate. After 24 h at 37 °C in a 5% CO_2_ atmosphere, cells were incubated for 24 h with different samples (Aβ_42_ ADDLs, Aβ_42_ + PrP_107–120_, Aβ_42_ ADDLs + PrP_107–120_ and PrP_107–120_, all pre-dissolved and pre-incubated in 2% (*v*/*v*) DMSO and F-12 Ham medium, as described above). Final concentrations of Aβ_42_ and PrP_107–120_ in DMEM were 3 and 0.75 µM (m.e.), respectively, with a 4:1 molar ratio. Cells were incubated with 0.5 mg/mL of MTT solution in Roswell Park Memorial Institute (RPMI) medium for 3 h at 37 °C, then with lysis buffer (20% SDS, 50% n,n-dimethylformamide, pH 4.7) for 1 h at 37 °C. The optical density was measured at 590 nm by a microplate reader (BioTek, Winooski, VT, USA).

### 4.11. Measurement of Intracellular Ca^2+^ Levels

SH-SY5Y cells were seeded on a glass coverslip in a six-well plate at a density of 40 × 10^3^ cells per well. After 24 h, 600 µL of various samples (Aβ_42_ ADDLs, Aβ_42_ + PrP_107–120_, Aβ_42_ ADDLs + PrP_107–120_ and PrP_107–120_, all pre-dissolved and pre-incubated in 2% (*v*/*v*) DMSO and F-12 Ham medium, as described above) were added to cells for 1 h at 37 °C. Final concentrations of Aβ_42_ and PrP_107-120_ in DMEM were 3 and 0.75 µM (m.e.), respectively. After washing with phosphate buffered saline (PBS), cells were loaded with a 4-µM Fluo-4 AM probe (Invitrogen/Thermo Fisher Scientific, Waltham, MA, USA) for 10 min at 37 °C. Imaging was performed after excitation at 488 nm with a TCS SP8 confocal scanning microscopy system (Leica Microsystems, Mannheim, Germany), using a Leica Plan Apo 63x oil immersion objective, taking a series of 1-µm-thick optical sections (1024 × 1024) through the cell depth for each sample and projecting them as a single composite image by superimposition. A minimum of four images were captured for each sample and four replicates were used for each condition. Images were analyzed using Image J software (NIH, Bethesda, MD, USA).

### 4.12. Measurement of Intracellular Reactive Oxygen Species (ROS)

To detect intracellular accumulations of ROS, SH-SY5Y cells were cultured for 24 h on glass coverslips in a 6-well plate at a density of 40 × 10^3^ cells per well. The medium was then replaced with 600 µL of various samples (Aβ_42_ ADDLs, Aβ_42_ + PrP_107–120_, Aβ_42_ ADDLs + PrP_107–120_ and PrP_107–120_, all pre-dissolved and pre-incubated in 2% (*v*/*v*) DMSO and F-12 Ham medium, as described above) with final concentrations of 3 and 0.75 µM (m.e.) for Aβ_42_ and PrP_107–120_ in DMEM, respectively. After 45 min, 5 µM of 2,7-dichlorodihydrofluorecein diacetate probe (CM-H2DFDA, Thermo Fisher Scientific, Waltham, MA, USA) was added for 15 min at 37 °C. Finally, cells were washed twice in PBS and then fixed in 2% (*w*/*v*) paraformaldehyde for 10 min at room temperature. Cell image acquisition was performed using the TCS SP8 confocal system described in Section 4.11. All measurements were performed in triplicates and ROS levels were calculated with Image J software (NIH). The ROS detected with this probe included mainly hydrogen peroxide [70].

### 4.13. Immunofluorescence Staining

SH-SY5Y cells were seeded in glass coverslips for 24 h in a 6-well plate at a density of 40 × 10^3^ cells per well, then exposed to 600 µL of various samples (Aβ_42_ ADDLs, Aβ_42_ + PrP_107–120_, Aβ_42_ ADDLs + PrP_107–120_ and PrP_107–120_, all pre-dissolved and pre-incubated in 2% (*v*/*v*) DMSO and F-12 Ham medium, as described above) with final concentrations of 3 and 0.75 µM (m.e.) for Aβ_42_ and PrP_107-120_ in DMEM, respectively. After 1 h at 37 °C, cells were washed with PBS and stained with 1:1000 diluted Alexa Fluor 633-conjugated wheat germ agglutinin (Life Technologies/Thermo Fisher Scientific, Waltham, MA, USA) for 15 min. Cells were rinsed again and fixed with 2% (*w*/*v*) paraformaldehyde for 10 min at room temperature. After cell membrane permeabilization with 0.5% BSA in PBS + 0.5% Triton X-100, cells were incubated with 1:800 diluted mouse monoclonal 6E10 antibody (BioLegend, San Diego, CA, USA) for 1 h in 37 °C. After washing three times with PBS, cells were incubated with 1:1000 diluted Alexa Fluor 488-conjugated anti-mouse secondary antibody (Life Technologies/Thermo Fisher Scientific, Waltham, MA, USA) for 1 h. All experiments were repeated three times and representative images of confocal microscopy are presented. Fluorescence was quantified with Image J software (NIH).

### 4.14. Dot Blot

To determine the effect of PrP_107–120_ on ADDLs structure, 2 µL of each sample (Aβ_42_ ADDLs, Aβ_42_ + PrP_107–120_, Aβ_42_ ADDLs + PrP_107–120_ and PrP_107–120_, all dissolved in 2% (*v*/*v*) DMSO and F-12 Ham medium, as described above) were transferred onto a nitrocellulose membrane and allowed to dry for 15 min. For blocking the membrane, 1% (*w*/*v*) BSA in Tris-buffered saline and 0.1% Tween 20 (TBST) was used. After 1 h, the membrane was probed with 1:800 diluted mouse monoclonal antibody 6E10 (BioLegend, San Diego, CA, USA) or with 1:500 human antibody specific to ADDLs (clone 19.3, Creative Biolabs, Shirley, NY, USA) at 4 °C overnight. The day after, the membrane was washed three times and subsequently incubated for 1 h at room temperature with 1:3000 peroxidase-conjugated anti-mouse secondary antibody (Abcam, Cambridge, MA, USA) or 1:1000 peroxidase-conjugated anti-human secondary antibody (EMD Millipore, Temecula, CA, USA). Imaging was performed using an Amersham imager 600 (Cytiva, Washington DC, MD, USA).

### 4.15. ANS Binding Assay

The ANS solution was prepared by dissolving 30 mg of 8-anilino-1-naphthalenesulfonic acid (ANS, Sigma-Aldrich) in 10 mL of 25-µM phosphate buffer, with a pH of 6.0. After 20 min shaking, the solution was filtered by a 0.45-µm filter. ANS concentration was measured by optical absorbance at 375 nm and diluted in the same buffer to 55 µM. For each experiment, 450 µL of ANS solution was mixed with 50 µL of each sample (Aβ_42_ ADDLs, Aβ_42_ + PrP_107–120_, Aβ_42_ ADDLs + PrP_107–120_ and PrP_107–120_, all dissolved in 2% (*v*/*v*) DMSO and F-12 Ham medium, as described above) or buffer as a blank. A 2- × 10-mm optical path-length was used and fluorescence spectra were recorded at 25 °C using the same PerkinElmer LS 55 fluorimeter described above, with excitation at 380 nm and emission at 400–650 nm.

### 4.16. Analysis of ADDLs Co-Localization with PrP_107-120_

BODIPY TMR-X NHS Ester (Thermo Fisher Scientific, Waltham, MA, USA) was dissolved in DMSO. PrP_107–120_ was dissolved in 0.1 M NaHCO_3_ buffer, with a pH of 7.0. The two solutions were diluted at room temperature with continuous shaking for 1 h in the latter buffer at final concentrations of 3 mM peptide and 0.3 mM dye. Cells were cultured on glass coverslips in a 6-well plate at a density of 40 × 10^3^ cells per well. After 24 h, cells were treated for 1 h with 600 µL of various samples (Aβ_42_ ADDLs, Aβ_42_ + labelled PrP_107–120_, Aβ_42_ ADDLs + labelled PrP_107–120_ and labelled PrP_107–120_, all pre-dissolved and pre-incubated in 2% (*v*/*v*) DMSO and F-12 Ham medium, as described above) with final concentrations of 3 and 0.75 µM (m.e.) for Aβ_42_ and labelled PrP_107–120_ in DMEM, respectively. After rinsing with PBS twice, 1:100 diluted fluorescent dye Hoechest (Immunochemistry Technologies, Bloomington, MN, USA) was added for 10 min at 37 °C. Following four washing steps with PBS, cells were fixed using 2% paraformaldehyde. Permeabilization of cells and Aβ_42_ staining were performed as described above. In another experiment, the concentration of labelled PrP_107–120_ was increased to 100 µM, and a 1:1 ratio for labelled PrP_107–120_ to Aβ_42_ was used in the initial pre-treatment in 2% (*v*/*v*) DMSO and F-12 Ham medium to prepare the Aβ_42_ + labelled PrP_107–120_ sample.

### 4.17. Analysis of PrP_107-120_ Co-Localization with PrP^C^

PrP_107-120_ was labelled with BODIPY TMR-X NHS Ester, as described in Section 4.16. SH-SY5Y cells were cultured on a glass coverslip in a 6-well plate for 24 h at a density of 40 × 10^3^ cells per well. After washing with PBS, cells were treated with 600 µL of labelled peptide at a final concentration of 0.75 µM for 1 h. After washing with PBS twice, 2% paraformaldehyde was added for 10 min. After fixation, cells were incubated with 1:250 diluted mouse monoclonal PrP (5B2) antibody (Santa Cruz Biotechnology, Santa Cruz, CA, USA) for 1 h at 37 °C. After washing with PBS, cells were incubated with 1:1000 diluted Alexa Fluor 488-conjugated anti-mouse secondary antibody (Life Technologies/Thermo Fisher Scientific, Waltham, MA, USA). Imaging was performed after excitation at 488 and 561 nm to detect the cellular prion protein and the PrP_107–120_ peptide, respectively, using the TCS SP8 confocal system described in Section 4.11.

### 4.18. Statistical Analysis

All data are presented as means ± S.E.M. (standard error of the mean). The difference between groups was analyzed using a Student’s t-test. The single (*/#), double (**/##) and triple (***/###) symbols refer to *p* values < 0.05, < 0.01 and < 0.001, respectively.

## Abbreviations

AD	Alzheimer’s disease
PrPC	Cellular prion protein
Aβ	Amyloid beta
LTP	Long-term potentiation
GPI	Glycosyl-phosphatidyl-inositol
R_h_	Hydrodynamic radius
D_h_	Hydrodynamic diameter
ADDLs	Amyloid-derived diffusible ligands
DMSO	Dimethyl sulfoxide
HFIP	Hexafluoro-2-isopropanol
ThT	Thioflavin T
CD	Circular dichroism
DLS	Dynamic light scattering
DMEM	Dulbecco’s Modified Eagle’s Medium
FBS	Fetal bovine serum
MTT	3-(4,5-dimethylthiazol-2-yl)-2,5-diphenyltetrazolium bromide
ROS	Reactive oxygen species
RPMI	Roswell Park Memorial Institute
BSA	Bovine serum albumin
BCA	Bicinchoninic acid
